# Macrosomic Neonates Carry Increased Risk of Dental Caries in Early Childhood: Findings from a Cohort Study, the Okinawa Child Health Study, Japan

**DOI:** 10.1371/journal.pone.0133872

**Published:** 2015-07-24

**Authors:** Hiroshi Yokomichi, Taichiro Tanaka, Kohta Suzuki, Tomoki Akiyama, Okinawa Child Health Study Group, Zentaro Yamagata

**Affiliations:** 1 Department of Health Sciences, Division of Medicine, Graduate School Department of Interdisciplinary Research, University of Yamanashi, 1110 Shimokato, Chuo City, Yamanashi, Japan; 2 Department of Social Medicine, Faculty of Medicine, Toho University, 5-21-16, Ohmorinisi, Ohta Ward, Tokyo, Japan; University of Bremen, GERMANY

## Abstract

**Background:**

Although many studies have discussed health risks in neonates with a low birth weight, few studies have focused on the risks in neonates with a high birth weight. The objective of this study was to determine whether differences in the incidence of dental caries in early childhood are associated with birth weight status.

**Methods:**

A total of 117,175 children born in Okinawa Prefecture, Japan from 1997 to 2007 were included in this study. Medical professionals collected information about birth records, growth and development, parental child-rearing practices and dental health at 3 months, 18 months and 3 years of age. The risk of dental caries among neonates with macrosomia (birth weight ≥4000 g) was compared with that among neonates with normal weight (2500–3999 g). Sensitivity analyses included ‘large for gestational age’ (LGA, birth weight above the 90^th^ percentile for gestational age), which was relative to ‘appropriate for gestational age’ (birth weight between 10^th^ and 90^th^ percentiles). Relative risks and relative risk increases were estimated by multivariate Poisson regression.

**Results:**

At 3 years of age, the relative risk increases for dental caries after adjusting for confounding factors were 19% [95% confidence interval (CI), 11%–28%, P < 0.001] for macrosomic neonates and 12% (95% CI, 9%–16%, P < 0.001) for LGA neonates.

**Conclusion:**

Macrosomia and LGA were associated with an increased risk of dental caries in early childhood. Particular attention should be paid to abnormally large neonates.

## Introduction

Difficulties experienced during pregnancy and childbirth form the subject of on-going clinical and basic research worldwide. Children born to obese mothers exhibit increased rates of childhood obesity and subsequent paediatric lifestyle-related diseases;[1, 2] furthermore, a controlled maternal weight at birth reduces the risk of adulthood obesity.[3–6] Although numerous available studies have evaluated low birth weight as a risk factor for growth retardation, metabolic syndrome,[7] early puberty and attention-deficit hyperactive disorder (ADHD),[8] few have report the risks associated with macrosomia. Moreover, macrosomic infants are not carefully monitored.

The potential risk factors and preventive factors associated with paediatric dental caries include hypoplastic enamel,[9] early dental eruption, dental hygiene (brushing the teeth by parents, fluoridated water [10]), excess consumption of sweets,[11] prolonged breastfeeding,[12] prolonged nursing with a bottle,[13] irregular consumption of meals and snacks,[14] insufficient dietary calcium in both the mother and child, low parental socioeconomic status,[15] parental smoking[16, 17] and support for child rearing.[18] Although all of these environmental and genetic factors are potentially causative or preventative candidates in terms of caries, the neonatal size has not been considered in these studies, despite its potential impact on the child’s dental health.

Although considerable research has been undertaken to determine whether caries are associated with obesity, previous reports have been unable to end the controversy because of confounding factors.[19] In particular, the association between obesity in toddlerhood and caries in primary teeth remains inconclusive.[20] Similarly, whether a low birth weight is a protective or risk factor for dental caries remains controversial.[21, 22] Moreover, there are no published data on the incidence of dental caries among neonates with high birth weights. Only one cohort study in the United Kingdom reported a small linear relationship between the caries incidence and birth weights ranging from low to high (odds ratio = 1.08 per 100 g of birth weight); however, this relationship was not significant.[23] In the previous study, the number of enrolled children was limited to 985, and the applied linear regression analysis did not investigate the possible J-shaped caries risk from low to high birth weights. Therefore, we aimed to determine whether a high birth weight was associated with the incidence of dental caries in primary teeth in a large population, simultaneously considering many risk factors associated with caries.

## Methods

### Ethics statement

The Ethics Review Committee of the Faculty of Medicine of the University of Yamanashi approved the study protocol in accordance with the ethical guidelines and regulations of the Declaration of Helsinki. The Japanese guidelines permit the use of data from medical examinations without consent if the data are anonymous; therefore, informed consent was not required for the current investigation.

### Study design and participants

The Okinawa Child Study is a cohort study based on the free health examinations provided to children by administrative authorities.[24] More than 82% of the approximately 16,000 children born annually in Okinawa participate in these health examinations at 3 months, 18 months and 3 years of age.[25] The present study was conducted using data from participants born between 2 April 1997 and 1 April 2007 (i.e. Japanese school years from 1997 to 2006). In this study, data from children who lacked birth records, medical records or oral health records were excluded.

### Measurements

Data regarding birth records, family composition, parental child-rearing practices, dietary habits and dental examinations were collected from the child health examinations. We analysed data for the following variables: sex, birth weight, gestational age, parity, siblings, children’s dental hygiene, children’s history of dental fluoridation, use of a bottle for nursing, consumption of cow’s milk and snacks, parental age, parental occupation, parental smoking habits and people involved in child-rearing. Qualified public health nurses and paediatricians performed all examinations for determining parental child-rearing practices, anthropometrics, growth and development. Qualified dentists evaluated the children’s oral health and diagnosed dental caries. Each child underwent this dental examination conducted by an on-site dentist. During the paediatric health examinations, the public health nurses interviewed mothers regarding their child-rearing practices. Macrosomia, normal birth weight and low birth weight were defined as birth weights of ≥4000 g,[26–29] 2500–3999 g and <2500 g, respectively, in accordance with the standards of the World Health Organization.[30] The definitions of ‘large for gestational age’ (LGA), ‘appropriate for gestational age’ (AGA) and ‘small for gestational age’ (SGA) were weights above the 90^th^ percentile, between the 10^th^ and 90^th^ percentiles and below the 10^th^ percentile for gestational age, respectively.[31, 32] Herein, birth weight reference categories used to compare the risk of caries were normal birth weight and AGA.

### Statistical analysis

The risks of having caries at 3 years of age among neonates with macrosomia and low birth weights were compared with the risks in other neonates. Multivariate Poisson regressions were used to estimate the relative risks (RR) with respect to the controlled confounding factors, as odds ratios tend to misrepresent the risks of exposure for high prevalence or incidence.[33, 34] RRs were determined for the following explanatory variables: sex, birth weight, mother’s age, gestational age, birth order, the number of teeth at 18 months, parents’ employment status, use of a bottle for nursing at 18 months, dental fluoridation, siblings, parental smoking, brushing the teeth by parents at 18 months and 3 years of age, drinking cow’s milk at 18 months and 3 years of age, eating irregular meals and snacks at 18 months and 3 years of age and watching TV or videos. Although some literature suggests breastfeeding as a risk factor of dental caries,[35] as a cultural norm, Japanese paediatricians and domestic public health nurses encourage mothers to begin ablactating at 5–6 months and to finish by the time their child is 18 months age.[36] Therefore, breastfeeding and ablactating were not considered confounding factors for dental caries at 3 years of age. Regarding the use of pacifiers, the Japanese Society of Pediatric Dentistry and Japanese mothers are aware of the risks of interrupted normal bite resulting from pacifier use.[37] As a result, the proportion of pacifier use is low; therefore, we did not consider it to be a confounding factor in Japan. Relative risk increases (RRI) were calculated as RR − 1. Furthermore, we calculated the adjusted least square estimates of the number of decayed or filled teeth to assess the marginal means over a population that had been adjusted according to other explanatory variables in a multivariable regression. Descriptive statistical analyses and estimations of RRs were performed using SAS statistical software (version 9.3, SAS Institute, Cary, NC, USA). Descriptive statistics are reported as means and standard deviations (SDs), and point estimates are reported with 95% confidence intervals (CI). All reported P values are 2-sided, and a P-value <0.05 was considered statistically significant.

### Sensitivity analysis

The United States Preventive Service Task Force and American Academy of Pediatrics recommend that fluoride varnish treatment should begin with primary tooth eruption.[38, 39] Accordingly, we believed that previous clinical fluoridation of primary teeth might have modified the risks, and therefore previous use of fluoride varnish was included in the first sensitivity analysis; this was restricted to the school years of 2006 and 2007, when detailed records of previous clinical fluoride varnish treatment were preserved. The second sensitivity analysis included LGA, AGA and SGA instead of macrosomia, normal birth weight and low birth weight, respectively; LGA, AGA and SGA were applied to the subjects who were evaluated according to the standards for Japanese children in the guidelines of Japan Obstetrics and Gynaecology.[32] In the third sensitivity analysis, the included children were restricted to those born through labour at term (i.e. gestational age of 37–41 weeks); accordingly, children born through pre- and post-term deliveries were excluded to minimize the result of exposure on the outcome, as gestational age may be associated with an earlier or later eruption of teeth susceptible to caries. Fourth, multivariate analyses of the association between macrosomia and dental caries were performed in the school years 1997–1999, 2000–2002 and 2003–2006 to explore how secular changes in diet contributed to the results. In all sensitivity analyses, multivariate Poisson regressions to adjust for confounding factors were conducted in the same manner used for the main analysis.

## Results

Among the 117,651 participants who underwent the health examination and for whom demographic data were available, we excluded 476 children who were toothless at 3 years of age, leaving a final sample size of 117,175. Table 1 shows the following mean demographic values among the population with 60,167 (51.4%) male subjects: birth weight, 3014 g (SD, 434); gestational age, 38.0 weeks (SD, 5.6); age of the mother at the 3-month examination, 29.7 years (SD, 5.6); birth order, 1.9 (SD, 1.0) and number of teeth at 18 months of age, 14.5 (SD, 2.7). Dental caries were present in 53,924 children (46.0%) at 3 years of age, and 1266 children (1.1%) were born with macrosomia.

**Table 1:** Background characteristics of children and their mothers in Okinawa, Japan.

Characteristics	Mean (SD) or number (%)
**Number of children**	**117,175**
**Number of male children**	**60,167 (51.4)**
**Birth weight, g**	**3014 (434)**
**Gestational age, weeks**	**38.0 (5.6)**
**Age of mother when child is 3 months, years**	**29.7 (5.6)**
**Order of birth**	**1.9 (1.0)**
**Number of teeth at 18 months**	**14.5 (2.7)**
**Number of children with caries at 3 years**	**53,924 (46.0)**
**Number of neonates born with macrosomia** ^**1**^	**1266 (1.1)**

^**1**^ **Macrosomia was identified as a birth weight ≥4000 g**

On univariate analysis, the risk of having caries at 3 years of age was significantly higher among children with macrosomia relative to those with normal birth weights (RRI, 23%, CI, 18–29, P < 0.001). Other univariate analyses yielded the following RRIs: 19% (CI, 17–21, P < 0.001) for a maternal age <25 years, 7% (CI, 0.01–14, P = 0.0496) for post-term delivery, 21% (CI, 19–22, P < 0.001) for non-firstborn babies, 14% (CI, 12–16, P < 0.001) for ≥14 teeth at 18 months of age, 22% (CI, 19–25, P < 0.001) for unemployed parents, 21% (CI, 19–23, P < 0.001) for maternal or paternal smoking, −5% (CI, −6–−3, P < 0.001) for no siblings at 3 years of age, 27% (CI, 23–31, P < 0.001) for no support for child rearing, 40% (CI, 38–42, P < 0.001) for occasional brushing the teeth by parents at 3 years of age, −19% (CI, −20–−18, P < 0.001) for drinking cow’s milk and 32% (CI, 30–34, P < 0.001) for irregular consumption of meals and snacks at 18 months of age (Table 2).

**Table 2:** Relative risk increases (RRIs) of developing caries among 3-year-old children in Okinawa, Japan by univariate and multivariate Poisson regressions. Relative risk increase equals relative risk minus 1.

Risk factors				Univariate analyses			Multivariate analysis			
		No. of children	Proportion of having caries at 3 years (%)	Crude RRI (%)	95% CI^3^	P value	Adjusted RRI (%)	95% CI^3^	P value	Adjusted no. of decayed and filled teeth at 3 years^4^
**Sex**	**Girls**	**57,008**	**44.7**	**Ref**	**––**	**––**	**Ref**	**––**	**––**	**2.79**
	**Boys**	**60,167**	**47.2**	**6**	**4–7**	**<0.001**	**3**	**2–5**	**<0.001**	**2.84**
**Birth weight (g)**	**2500–3999**	**104,442**	**46.1**	**Ref**	**––**	**––**	**Ref**	**––**	**––**	**2.65**
	**≥4000**	**1266**	**56.9**	**23**	**18–29**	**<0.001**	**19**	**11–28**	**<0.001**	**3.30**
	**<2500**	**11,467**	**44.1**	**−4**	**−6–−2**	**<0.001**	**−5**	**−8–−1**	**0.005**	**2.50**
**Age of mother (years)**	**25–34**	**74,344**	**43.9**	**Ref**	**––**	**––**	**Ref**	**––**	**––**	**2.64**
	**<25**	**20,091**	**52.1**	**18**	**17–21**	**<0.001**	**17**	**14–20**	**<0.001**	**3.08**
	**≥35**	**22,740**	**47.5**	**8**	**7–10**	**<0.001**	**2**	**0.1–5**	**0.04**	**2.72**
**Gestational age**	**Labour at term**	**106,409**	**45.8**	**Ref**	**––**	**––**	**Ref**	**––**	**––**	**2.76**
	**Pre-term delivery**	**9801**	**47.7**	**4**	**2–6**	**<0.001**	**3**	**−1–7**	**0.10**	**2.89**
	**Post-term delivery**	**965**	**48.9**	**7**	**0.01–14**	**0.0496**	**7**	**−3–18**	**0.21**	**2.80**
**Order of birth**	**first**	**50,354**	**41.1**	**Ref**	**––**	**––**	**Ref**	**––**	**––**	**2.53**
	**other**	**66,801**	**49.7**	**21**	**19–22**	**<0.001**	**26**	**24–29**	**<0.001**	**3.10**
**No. of teeth at 18 months**	**0–13**	**19,042**	**38.9**	**Ref**	**––**	**––**	**Ref**	**––**	**––**	**2.59**
	**14–20**	**56,059**	**44.3**	**14**	**12–16**	**<0.001**	**13**	**11–16**	**<0.001**	**3.04**
**Both parents are jobless at 3 years**	**No**	**111,984**	**45.6**	**Ref**	**––**	**––**	**Ref**	**––**	**––**	**2.58**
	**Yes**	**5191**	**55.6**	**22**	**19–25**	**<0.001**	**11**	**6–16**	**<0.001**	**3.05**
**Bottle use at 18 months**	**No**	**40,407**	**42.3**	**Ref**	**––**	**––**	**Ref**	**––**	**––**	**2.76**
	**Yes**	**34,668**	**44.3**	**5**	**3–7**	**<0.001**	**4**	**2–6**	**<0.001**	**2.87**
**Experience of dental fluoridation at 3 years** ^**1**^	**Yes**	**8023**	**35.8**	**Ref**	**––**	**––**	**––**	**––**	**––**	**––**
	**No**	**8195**	**37.5**	**2**	**−2–7**	**0.07**	**––**	**––**	**––**	**––**
**Maternal or paternal smoking at 3 years**	**No**	**51,352**	**40.9**	**Ref**	**––**	**––**	**Ref**	**––**	**––**	**2.64**
	**Yes**	**51,298**	**49.5**	**21**	**19–23**	**<0.001**	**15**	**13–17**	**<0.001**	**2.99**
**Sibling <6 years at 3 years** ^**2**^	**No**	**54,062**	**47.2**	**Ref**	**––**	**––**	**––**	**––**	**––**	**2.85**
	**Yes**	**63,113**	**45**	**−5**	**−6–−3**	**<0.001**	**––**	**––**	**––**	**2.78**
**Someone who supports child rearing at 3 years**	**Yes**	**6564**	**36.7**	**Ref**	**––**	**––**	**Ref**	**––**	**––**	**2.63**
	**No**	**110,529**	**46.6**	**27**	**23–31**	**<0.001**	**17**	**13–22**	**<0.001**	**3.00**
**Brushing the teeth by parents at 18 months**	**Daily**	**41,668**	**38.4**	**Ref**	**––**	**––**	**Ref**	**––**	**––**	**2.60**
	**Sometimes/never**	**33,589**	**49.3**	**29**	**26–31**	**<0.001**	**18**	**16–20**	**<0.001**	**3.03**
**Brushing the teeth by parents at 3 years**	**Daily**	**101,153**	**43.6**	**Ref**	**––**	**––**	**Ref**	**––**	**––**	**2.42**
	**Sometimes/never**	**13,867**	**61**	**40**	**38–42**	**<0.001**	**22**	**19–25**	**<0.001**	**3.21**
**Drinking cow’s milk at 18 months**	**No**	**68,864**	**49.9**	**Ref**	**––**	**––**	**Ref**	**––**	**––**	**3.04**
	**Yes**	**48,311**	**40.5**	**−19**	**−20–−18**	**<0.001**	**−12**	**−14–−10**	**<0.001**	**2.59**
**Drinking cow’s milk at 3 years**	**No**	**41,593**	**49.3**	**Ref**	**––**	**––**	**Ref**	**––**	**––**	**2.85**
	**Yes**	**75,582**	**44.2**	**−10**	**−12–−9**	**<0.001**	**−5**	**−6–−3**	**<0.001**	**2.78**
**Irregular meals and snacks at 18 months**	**No**	**54,088**	**39.8**	**Ref**	**––**	**––**	**Ref**	**––**	**––**	**2.55**
	**Yes**	**20,487**	**52.4**	**32**	**30–34**	**<0.001**	**16**	**13–18**	**<0.001**	**3.08**
**Irregular meals and snacks at 3 years**	**No**	**73,838**	**41.6**	**Ref**	**––**	**––**	**Ref**	**––**	**––**	**2.58**
	**Yes**	**39,345**	**54**	**30**	**28–31**	**<0.001**	**16**	**14–19**	**<0.001**	**3.05**
**TV or video watching every day at 3 years** ^**1**^	**No**	**1964**	**43.2**	**Ref**	**––**	**––**	**––**	**––**	**––**	**––**
	**Yes**	**2355**	**44.8**	**4**	**−3–11**	**0.30**	**––**	**––**	**––**	**––**

^1^These variables were eliminated from the multivariate analysis because many values were missing.

^2^This variable was eliminated from the multivariate analysis to avoid multicollinearity.

^3^Confidence interval

^4^Least square estimates in a multivariate regression calculated adjusted number of decayed and filled teeth at 3 years of age.

The following RRIs were obtained through the main multivariate regression: 19% (CI, 11–28, P < 0.001) for macrosomic babies, 17% (CI, 14–20, P < 0.001) for a maternal age <25 years, 7% (CI, −3–18, P = 0.21) for post-term delivery, 26% (CI, 24–29, P < 0.001) for non-firstborn babies, 11% (CI, 6–16, P < 0.001) for unemployed parents, 15% (CI, 13–17, P < 0.001) for maternal or paternal smoking, 17% (CI, 13–22, P < 0.001) for no support for child rearing, 22% (CI, 19–25, P < 0.001) for occasional brushing the teeth by parents at 3 years of age, −12% (CI, −14–−10, P < 0.001) for drinking cow’s milk and 16% (CI, 13–18, P < 0.001) for irregular consumption of meals and snacks at 18 months of age (Table 2).

The first sensitivity analysis, which was adjusted for previous clinical fluoride varnish treatment, estimated the RRIs for macrosomic birth weight and the effect of fluoridation on the incidence of caries at 3 years of age to be 4% (CI, −17–30, P = 0.72) and 4% (CI, −2–9, P = 0.16), respectively. In the second sensitivity analysis, the estimated RRIs for LGA and SGA were 12% (CI, 9–16, P < 0.001) and −1% (CI, −4–2, P = 0.49), respectively. In the third sensitivity analysis, which was restricted to children born via labour at term, the RRI of a macrosomic birth weight for caries at 3 years of age was 19% (CI, 11–28, P < 0.001). In the fourth sensitivity analysis, the estimated RRIs for macrosomia were 20% (CI, 6–36, P < 0.01) during 1997–1999, 17% (CI, 4–32, P < 0.01) during 2000–2002 and 16% (CI, 3–30, P = 0.01) during 2003–2007.

## Discussion

### Main findings

Among macrosomic children, the RRI of caries at 3 years of age was 19%, representing a significant increase relative to children with normal birth weights. Four sensitivity analyses, which considered the clinical use of fluoride varnish, LGA, labour at term and secular trends, yielded approximately the same RRIs for children with high birth weights.

### Possible reasons for these associations

Although the mechanism underlying the formation of caries after exposure to intrauterine over-nutrition is not understood, relatively high concentrations of glucose and amino acids in utero may increase the postnatal appetite or insulin secretion. Evidence indicates that overweight pregnant women have high concentrations of inflammatory cytokines and increased insulin resistance,[40] and subsequently compensatory hyperinsulinaemia and foetal adiposity occur in utero.[41, 42] Hoegsberg et al. observed that macrosomic neonates of nondiabetic mothers are more likely to exhibit hyperinsulinaemia than normal size neonates.[43] Moreover, in foetal rhesus monkeys, external insulin injection resulted in the delivery of macrosomic neonates with hyperinsulinaemia.[44] Dörner et al. suggested that perinatal hyperinsulinaemia alters the function of hypothalamic ventromedial nuclei, which play a determining role in satiety, appetite and insulin secretion from pancreatic β-cells.[45] Studies in rats have revealed foetal β-cell hyperplasia in the offspring of moderately diabetic mothers.[46, 47] The ‘Developmental over-nutrition hypothesis’, which was recently submitted based on animal model studies, proposes that maternal nutritional and hormonal conditions during pregnancy programme the appetite and energy expenditure of the offspring, as well as the hormonal, neuronal and autocrine mechanisms that contribute to the offspring’s energy balance.[48] Thereafter, the primary teeth of LGA children may be more frequently exposed to cariogenic food and drink than the teeth of AGA children. Furthermore, the finding that LGA neonates remain classified as overweight until the age of 83 months[49, 50] might reflect the hypothetical programming in utero and subsequent voracious appetite.

### Comparison with other studies

Regarding the positive effect of macrosomia on dental caries, we have calculated the following: if the previous report from the UK regarding a small positive association between birth weight and the risk of caries (odds ratio (OR) = 1.08 per 100 g of birth weight)[23] could be directly applied to 4000 g (lower limit of macrosomia) with a reference of, for instance, 3000 g, the OR may be calculated as 2.16 [i.e. 1.08 to the 10^th^ power, where 10 equals (4000−3000)/100]; the calculated OR is mathematically equivalent to a RR of 1.41 and RRI of 41%, given that the risk of caries was 46% among children born at a weight of 3000 g. Therefore, our estimated RRI = 19% was not overly large, and we consider our result to be consistent with the previous study. Regarding the negative RRI of −5% for a low birth weight, our results were similarly consistent with the above-described report from the UK. In addition, Saraiva et al. suggested that SGA, foetal growth restriction and pre-term birth should be associated with a lower incidence of dental caries among Caucasian, African-American and Mexican-American children aged 2–5.9 years.[51] Our results regarding low birth weight and SGA provided evidence from a Japanese population to support the findings of Saraiva et al. A programmed lower appetite, as discussed above, might explain these observations related to the lower incidence of caries in children with low birth weights.

### Sensitivity analyses

In the first sensitivity analysis, which was restricted to data from 2 school years and included the clinical use of fluoride varnish, the RRI adjusted for previous fluoride varnish use was 4% (CI, −17–30, P = 0.72), with a lower result in the main multivariate analysis. Taking the large CI and non-significance into account, we considered that these data related to fluoride varnish use were unreliable for estimating the effect of macrosomia. In other words, in an era when almost all commercial fluoridated dental toothpastes contain fluoride and dental clinics and individual dentists routinely apply fluoride rinses, gels or varnishes to the primary teeth during paid clinical examinations, we do not consider previous fluoride varnish use to be an important confounding factor in the present study. The paradoxical effect of previous clinical fluoride varnish use, which has an RRI of 4% (CI, −2–9, P = 0.16), may represent the uselessness of this information in the present-day survey.

In the second sensitivity analysis, in which our exposure variable of interest was changed from rare macrosomia (birth weight ≥4000 g) to LGA, the RRI of LGA for caries development (12%) was similar to that of macrosomia (19%); in addition, the relatively lower RRI associated with LGA can be reasonably explained by the fact that LGA is, by definition, more frequent than macrosomia. It should be emphasized that the third sensitivity analysis, which excluded children born via pre- and post-term delivery and was therefore restricted to those born through labour at term, yielded the same RRI of 19%. Because the timing of tooth eruption and the number of susceptible teeth among term neonates are considered to be approximately the same, this sensitivity analysis confirmed our report regarding the effect of macrosomia on dental caries in early childhood. Moreover, the fourth sensitivity analysis confirmed the RRI of macrosomia in recent years as 16–20%.

### Strengths and limitations

To the best of our knowledge, this is the first report to raise an alarm over the risk of a high birth weight with respect to dental caries of the primary teeth. A study in the UK suggested a weak linear relationship between incidental caries and birth weights ranging from low to high,[23] but that previous study could not clarify the risk of macrosomia by its linear regression analysis. In this study, we used birth weights as a categorical variable to examine whether both high and low birth weights are risk factors for dental caries (J-shaped risk); however, the results were not similar to those of the previous study. Second, our large amount of data obtained from the free health service provided to resident children by the administrative authorities retained sufficient statistical power to determine the risks of a high birth weight, whereas the data set in the previous study in the UK also lacked statistical power. Third, the sensitivity analyses confirmed the postulated risk of macrosomia. Furthermore, the measurements were valid because all of the health professionals were qualified to evaluate caries, anthropometrics and interviews addressing parental child-rearing practices and several socioeconomic statuses.

One limitation of this study was the lack of available information about parental dental hygiene and typical socioeconomic status. Parental self-dental care may have directly affected the incidence of caries in their children. However, because parental lifestyles and dental hygiene are reflected by the socioeconomic status, this potential confounding bias might have been reduced by adjusting for information regarding the parental employment status, smoking habits, siblings, child-rearing support provided by others, parental support for brushing their children’s teeth and irregular consumption of meals and snacks. Another limitation in interpreting the results would be that the dental caries were neither validated by other dentists nor validated for different periods of time. Owing to the nature of administrative massive health examinations, the number of on-site dentists is limited. Because there is no published data describing the likelihood of dental caries diagnosis at health examination centres compared to that at dental clinics, the influence of lack of validation on the number of diagnosed caries in this report is unknown. However, even if a misclassification of children with and without caries existed, dentists were unlikely to diagnose caries in view of child birth weights; therefore, we believe that the bias because of this influence in the reported RRIs would be minimal. The other potential limitation of this study is its regional restriction to Okinawa; therefore, the results may not be applicable to other regions of Japan. Moreover, during the study period, approximately 25% of children in Okinawa did not undergo health examinations or were excluded from the study because of insufficient information. It is possible that these missing children had poor access to health examinations and were born to families with low socioeconomic statuses. As a result, the frequencies of high birth weight and caries in that population might have exceeded those of the children studied, and accordingly the missing data might have biased the RRIs of our results.

### Implications and conclusions

To the best of our knowledge, we are the first to report that macrosomia significantly increases the risk of dental caries. Our focus on the influence of high birth weight on this paediatric lifestyle-related disease represents a new perspective in perinatal research. The mechanism of the relationship between macrosomia and dental caries remains under debate, and further laboratory and clinical studies are warranted. We recommend that children with macrosomia should receive close attention from medical professionals during development.
